# Treatment of Hemorrhoids and Fissue of the Anus

**Published:** 1886-08

**Authors:** K. P. Moore

**Affiliations:** Macon, Ga.


### TREATMENT OF HEMORRHOIDS AND FISSURE OF THE ANUS.

REMARKS MADE BEFORE THE MACON MEDICAL SOCIETY BY
K. P. MOORE, M. D., MACON, GA.

Reported for THE ATLANTA MEDICAL AND SURGICAL JOURNAL.

On the subject of hemorrhoids Dr. Moore said: Without going back for a cause of hemorrhoidal tumors, and coming directly to their management, the question which first presents itself is as to the *best* plan of treatment. This, of course, depends upon the age and character of the trouble. If the tumors are acute, or of recent date, perhaps the plan of making a simple incision through them is as good as any. If the tumors are of longer standing and are not filled with coagula of venous blood, he prefers the ligature to any other plan, and thinks it the most satisfactory and perfect in its results; that is to say, leaves no tags or other surplus tissue as a source of subsequent annoyance. The plan of injecting these tumors with carbolic acid has been a very popular one with the unprofessional pile-doctor, and has enabled him to promise with considerable assurance "to cure piles without the knife or ligature." Perhaps on this account it has never grown into very popular favor with the regular profession. The opinion prevails among the profession that this is a more hazardous operation than the others, but so far as I know statistics are wanting to prove this to be true. This plan, I have no question, has suffered at the hands of quacks and unqualified operators, and the apparent larger mortality rate may be due to a want of skillful application of the remedy. I have frequently employed this plan, and have never had an untoward symptom to result; nor have I ever failed to remove the tumor. It is not more painful than the knife or ligature, and cures by sloughing about as speedily as either of them. In chronic hemorrhoids, and especially where there is a surplus of mucous membrane, producing considerable prolapsus, no remedy with me is comparable to the ligature. I take up a tumor, with as much of the surplus membrane as I think proper, and pass through it the double-threaded needle and tie it on either side, tight enough, of course, to strangulate, and in a few days it sloughs off. Even where there is no well-developed tumor, but where there is a large amount of the rectum prolapsed, and in a state of chronic inflammation, so that it becomes a source of constant bleeding and nervous irritation, a perfect cure can be accomplished by ligating considerable portions of this prolapsed membrane and allowing it to slough off, and in this way get rid of it, and otherwise stimulate the remaining portion to healthy activity and return to natural condition. The actual cautery suggests itself to me as a good remedy in this condition, used upon the same principle as the ligature; that is, by applying to patches and not to the whole prolapsed mass; but my experience with this plan does not authorize me to speak confidently of it.

Mr. President, before leaving the rectum, I wish to speak of an exceedingly troublesome and painful affection, which, under ordinary management, has been quite intractable and hard to cure; and indeed I think it is questionable whether any amount of local or constitutional medication would ever effect a cure in a case of long standing. I refer to fissure of the anus. I know of no affection more painful, or calculated to produce more nervous purturbation than a fissured anus, especially where it has existed for a considerable time. The older the fissure the more irritable becomes the sphincter muscles, and the more irritable the sphincters become the more intractable does the disease become of cure. The irritable and constant activity of the sphincters develop along their course, little clumps or bellies of muscular fibers, and these in turn, stimulated by the pain, only make the muscles more active and contractile, and in this way the irritation and fissure is kept aflame and aglow in spite of all effort at treatment. Luckily the management and cure of these troublesome affections has been simplified and made easy and certain. It is simply to stretch the sphincters until these fibrous clumps are felt to give way, and the muscles are well stretched and, temporarily, partially paralyzed. It would be useless to undertake the operation without an anæsthetic—the patient could not endure the pain, nor could the operation be done successfully.

I undertook the operation only a few days ago without anæsthesia, and made a complete failure. Having anæsthetized the patient I introduce the two thumbs into the anus, and with the hands and fingers extended over each buttock make gradual but continued pressure until the sphincters are well dilated and the muscular clumps are felt to break up and give way. The patient may then be turned loose, and in a large majority of cases will require nothing more. I have never failed to give entire and permanent relief by this very simple operation. The first case of this sort treated by myself since I came to Macon was two years or more ago, and up to this time he remains entirely well. Dr. H. J. Williams kindly assisted me in this case. The patient had almost become a wreck, and was fast falling into the morphine and whisky habit from pain. He never took another dose of morphine after the operation, and fattened up like a pig. Two others have since come under my observation with similar satisfactory results. I gave no other treatment in either case.

